# Preparation of a Novel SERS Platform Based on Mantis Wing with High-Density and Multi-Level “Hot Spots”

**DOI:** 10.3390/nano9050672

**Published:** 2019-05-01

**Authors:** Mingli Wang, Guochao Shi, Junlin Zhu, Yanying Zhu, Xin Sun, Peng Wang, Tifeng Jiao, Ruifeng Li

**Affiliations:** 1 State Key Laboratory of Metastable Materials Science & Technology and Key Laboratory for Microstructural Material Physics of Hebei Province, School of Science, Yanshan University, Qinhuangdao 066004, China; wml@ysu.edu.cn (M.W.); yyzhu@ysu.edu.cn (Y.Z.); sunxin@ysu.edu.cn (X.S.); liruifeng910321@163.com (R.L.); 2 Hebei Key Laboratory of Applied Chemistry, School of Environmental and Chemical Engineering, Yanshan University, Qinhuangdao 066004, China; ysu_zjl@163.com; 3 State Key Laboratory of Metastable Materials Science and Technology, Yanshan University, Qinhuangdao 066004, China

**Keywords:** SERS, mantis wing, Ag nanofilm, Au NPs, hot spots

## Abstract

The recent development of SERS substrates based on irregular nanostructures for directly molecule recognition has aroused increasing attention. By combining the irregular flake-like nanostructures of mantis wings, high SERS performance of Ag nanofilms, and the chemical stability of Au nanoparticles (NPs), an ultra-sensitive and flexible SERS substrate based on Au NPs functionalized Ag nanofilms-mantis wings (Au-Ag-M.w.) hybrid system is successfully fabricated. When 4-aminothiophenol is selected as the probe molecule, the limit of detection (LOD) is as low as 10^−13^ M and the relative standard deviation (RSD) is lower than 7.15%. This novel SERS platform exhibits high SERS performance in terms of sensitivity, reproducibility and practicability mainly because there are high-density and multi-level “hot spots” in the appropriate nanogaps. Meanwhile, it also systematically compares the differences of the SERS performance of Cu and Ag decorated M.w. hybrids and how these differences can alter their response. Moreover, the proposed substrate is employed to rapidly detect the pesticide residues on apple peels and the LOD for cypermethrin is estimated at 10^−10^ mg/mL. Therefore, this novel SERS substrate has great potential in rapid sampling of pesticide residues on real samples and expands the investigation to other natural materials for fabricating various SERS platforms.

## 1. Introduction

Raman scattering spectroscopy is a rapid, nondestructive and qualitative analysis tool which can provide vibrational and rotational information of a molecule [1]. Recent years have witnessed an escalating research interest in SERS technology [2,3,4]. Compared with the conventional Raman scattering spectroscopy, this highly surface-sensitive analytical technique can directly provide information of various target molecules at very low concentrations or even single molecule level. Due to the high-performance and label-free detection of the probe molecules, SERS has great application prospects in the fields of biosensing, food safety, environmental monitoring, etc. [5,6,7]. In addition to the high sensitivity, SERS technique possesses another advantage in the high enhancement of Raman signal intensity. It is widely accepted that there are two major mechanisms that contribute to SERS enhancement. One is chemical enhancement (CM), and this enhancement mechanism is mainly based on the transfer effect of dynamic charge between the analytes and plasmonic nanostructures [8]; the other, electromagnetic enhancement mechanism (EM), originating from the sharp increase of local electromagnetic field at appropriate nanostructural junctions (“hot spots”) excited by localized surface plasmon resonance (LSPR), can provide Raman signal over several orders of magnitude higher than the CM [9]. In principle, the dramatically enhanced Raman signals can be sharply magnified as higher as a factor of ${\left|E\right|}^{4}$ [10].

In general, coinage metals such as Au, Ag and Cu with specific plasmonic nanostructures are the most popular SERS platforms [11,12,13,14,15,16]. Particularly, noble metal Ag, due to its clear LSPR absorption in the spectral regions from visible spectrum to near-infrared light [17,18,19,20], has been designed as effective plasmonic nanostructures to realize highly sensitive SERS detection. In literature, diverse technologies to achieve high-performance plasmonic nanoarrays have been reported, such as nanosphere lithography [21], electron-beam lithography [22], focused ion-beam lithography [23], laser direct writing [24], casting and solidification process [25] etc. These nanostructures can provide abundant “hot spots” and excite a high LSPR effect, but the complex and time-consuming experimental processes, strict experimental conditions, low-throughput fabrication processes further limit the widespread application in real-life detection. Although many works have been done to overcome these disadvantages [26,27,28], the application prospect is not clear yet. In other words, the manageable, high-producing and low-cost synthesis of SERS substrates with large-scale plasmonic arrays is still a big challenge.

Recently, plenty of scientific researches have confirmed that the natural world possesses multifunctional nanomaterials and nanostructures which can be applied in SERS applications due to the unique surface characteristics and low-cost acquirement of biomaterials. Meanwhile, considerable research efforts have suggested that simply modified with noble metals, biomaterials such as insect wings and plant leaves show excellent SERS effect for simultaneous determination of multi-molecules. For example, Mu et al. reported a novel SERS platform based on Au NPs/butterfly wing (Morpho, Papilio and Ornithoptera) as flexible and adhesive SERS substrate. The high-performance SERS substrate was employed to detect 4-aminothiophenol and the LOD was as low as 10^−9^ M [29]. According to Lv’s work, the recyclable SERS platform with abundant nanogaps was designed based on the cicada wings (Cryptotympana atrata) and it was successfully applied in detection of pesticide residues [30]. By using a textured taro leaf (collected from Himachal Pradesh, India) as a template, Kumar’s group fabricated a flexible SERS sensor by decorating Ag on the PDMS (polydimethylsiloxane) substrate which was duplicated from a Taro leaf [25]. The results revealed that high sensitivity (10^−11^ M for malachite green) and well reproducibility of Raman signals were due to the super-hydrophobicity and flexible surface. That is to say the SERS performance can be enhanced by the hydrophobic surface. Specifically, when a droplet of probe molecule solution is placed on the hydrophobic nanomaterial, the droplet will form to be a near-spherical shape. After evaporation, the probe molecules are highly concentrated into a tiny area, resulting in high sensitivity. In addition to high sensitivity, excellent Raman enhancement and super-hydrophobicity, these biomaterial-based SERS substrates possess another advantage in flexibility. They offer advantages over traditional rigid substrates in their flexibility and can sense analytes on the surface of non-planar geometries. What’s more, they possess excellent mechanical strain resistance and can be easily tailored into any desired shapes and sizes. This point has been well demonstrated and analyzed by many reported flexible SERS substrates [31,32,33].

**Figure 1:**
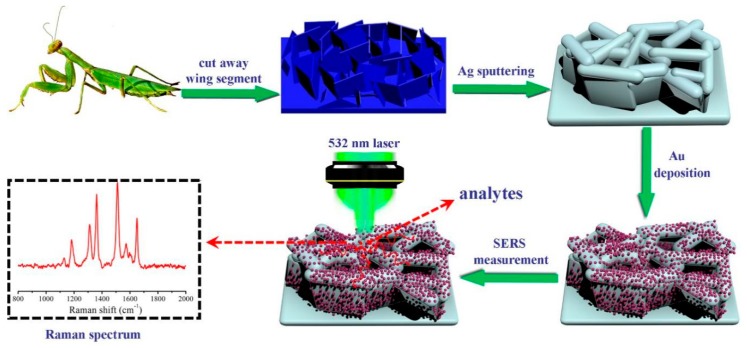
Schematic illustration of the fabrication process of the Au-Ag-M.w. nanoarray and SERS measurement by Raman system.

Fascinated by the flexible and hydrophobic bio-materials, we selected M.w. nanoarrays as bio-templates to fabricate an Au/Ag-decorated natural SERS substrate. As shown in Figure 1, the irregular flake-like nanoarray was first cleaned in deionized water. Afterward, the Ag nanofilms were sputtered on the M.w. by magnetron sputtering. Au NPs were decorated on the Ag-M.w. arrays via a simple physical deposition approach. Compared to the SERS-active substrates prepared by complex methods, our approach to fabricate the novel Au-Ag-M.w. substrate was facile and low-cost. Meanwhile, the Au NPs and Ag nanofilms on the densely packed nanoarray could provide the high-density and multi-level “hot spots”, thus ensuring high enhancement of Raman signals compared to the Cu-M.w. substrate reported previously [34]. Finally, due to the flexible surface, the Au-Ag-M.w.-20 (the sputtering time was 20 min) substrates were employed in the label-free detection of cypermethrin on apple peels via a direct and simple “contact scanning” method. The successful detection of cypermethrin on real samples indicates that the Au-Ag-M.w.-20 substrates show great potential for practical applications in the field of biochemical sensing and food monitoring.

## 2. Experimental Section

### 2.1. Materials and Instruments

Silver target (99.99%) was obtained from China Material Technology Co., Ltd. (Nanchang, China) M.w.s (Hierodula, Mantodea, Mantidae) were purchased from Beijing Jiaying Grand Life Sciences Co., Ltd. (Beijing, China). Experiments with mantis wings complied with the accepted ethical standards and were approved by the Ethical Review Board of Yanshan University on 15 January 2018. R6G, 4-aminothiophenol and cypermethrin were obtained from J&K Scientific Ltd. (Beijing, China). AuCl_3_·HCl·4H_2_O (99.8%) and Na_3_C_6_H_5_O_7_·2H_2_O (99.9%) were purchased from Tianjin Meizke Chemical Co., Ltd. (Tianjin, China). Deionized water (18.25 MΩ) was used for all solution preparations. 

Ag nanofilms were coated on the M.w.s by the DC magnetron sputtering system (DHRM-3) (Hangzhou Dahua instrument manufacturing Co. Ltd., Hangzhou, China). The surface morphology and size distribution of different substrates were characterized by field emission scanning electron microscopy (FE-SEM) (S-4800) (Hitachi, Tokyo, Japan). The morphology and size of Au NPs were characterized by Transmission Electron Microscope (TEM) (HT7700) (High-Technologies Corp., Ibaraki, Japan). Raman spectra of 4-aminothiophenol, R6G and cypermethrin were obtained by Raman system (inVia) (Renishaw, UK). The UV-vis absorption spectra were monitored by the UV-2550 UV-vis spectrophotometer (Shimadzu (China) Co., Ltd., Shanghai, China).

### 2.2. Sample Preparation

The M.w.s were dissected into approximately 1 × 1 cm^2^ and then cleansed by ethanol and deionized water for 10 min in turn to remove the organic contaminants. After natural drying, the Ag nanofilms were deposited. Sputtering process was carried in Argon gas with 99.9% purity and the vacuum degree of sputtering chamber was controlled at 2 × 10^−2^ mbar. The sputtering voltage was 90 V, the working current 170 mA. Au NPs were prepared by chemical reduction method according to modified Frens’s work [35]. The Au NPs were deposited on the Ag-M.w. substrate by physical deposition and dried in a vacuum drying oven. It should be pointed out that the terminologies to describe the samples of Ag-M.w. fabricated with the sputtering time of *x* min would be called Ag-M.w.-*x* substrate. Consistently, the Au-Ag-M.w.-*x* described these Ag-M.w.-*x* substrates decorated with Au NPs.

### 2.3. SERS Measurements

All the Raman spectra were obtained with a ×50 objective lens (NA = 0.75). For SERS measurements, the laser with the wavelength of 532 nm was used as the excitation source. The diameter of the laser spot was ~1 μm and the spectral resolution was about 1 cm^−1^. Meanwhile, the power of the laser was set at 0.1 mW to avoid structural changes due to laser heating. The diffraction grating was used 1800 lines/mm grating and the focal length was 250 mm. A 4-aminothiophenol stock solution of the concentration of 0.1 M has been used for further dilution from 10^−2^ M to 10^−13^ M. The 4-aminothiophenol aqueous solution (10 µL) was dropped on each fabricated SERS substrate prior to the SERS measurement, and dried in a vacuum drying oven at 40 °C for 5 min. The Raman spectra were recorded with an integrated accumulation time of 10 s. When dealing with the Raman data, a background removal algorithm was adopted, and the Raman spectra were smoothed to remove the disorderly peaks. In order to obtain a statistical result and reduce the influence caused by the laser, the Raman spectra were taken at five different areas of each sample and the collected Raman data were averaged.

### 2.4. Detection of Pesticide Residues on Apple Peels

The trace detection of cypermethrin on apple peels was followed Liu et al.’s method [36]. First, an apple was totally cleaned with deionized water and ethanol in order to remove the contaminants on the surface. After the evaporation, the thin peels were taken from apple and cut into uniform squares of 1 × 1 cm^2^. Subsequently, 10 μL different concentrations of cypermethrin solutions were separately dropped onto the peel samples with a pipette. After natural drying, 10 μL ethanol solution was added onto the peels to extract the pesticide residues and increase the concentration of cypermethrin molecules at the outer surface. The next step was to press the Au-Ag-M.w.-20 substrate to the treated sites and remained until completely dry and then peeled off for further analysis. In order to ensure the successful acquisition of Raman signals of cypermethrin, the measurements were performed five times randomly on the treated peels and the collected Raman data were averaged.

## 3. Results and Discussion

### 3.1. Comparing the SERS Substrates

**Figure 2:**
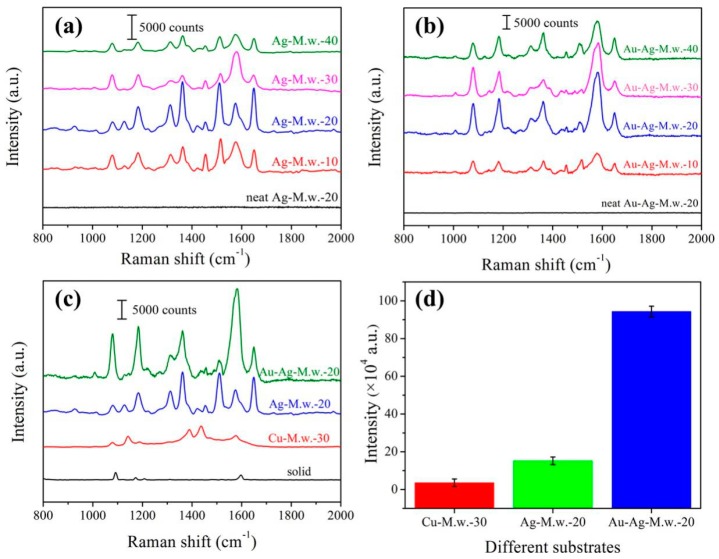
Raman spectra of 10^−4^ M 4-aminothiophenol on Ag-M.w.-*x* (**a**) and Au-Ag-M.w.-*x* substrates (**b**) prepared with different sputtering time and corresponding neat substrate spectra without 4-aminothiophenol absorbed; (**c**) Raman spectra of 10^−4^ M 4-aminothiophenol recorded from Cu-M.w.-30, Ag-M.w.-20 and Au-Ag-M.w.-20 substrate, respectively; (**d**) Comparison of Raman signal intensities at 1578 cm^−1^ of different substrates.

In our previous report [34], we have successfully fabricated the Cu decorated M.w. SERS substrate. On this basis, in order to further improve the sensitivity, SERS enhancement and stability of M.w.-based SERS substrates, we chose silver material with the best response to electromagnetic enhancement as sputtering targets, and a series of Ag decorated M.w. substrates with different sputtering time were discussed. In order to evaluate the SERS performance of the Ag-M.w.-*x* substrates, 4-aminothiophenol, which has been widely applied in SERS detection, was chosen as the probe molecule due to its well established vibrational features. Figure 2a exhibits the Raman spectra of 10^−4^ M 4-aminothiophenol recorded from different Ag-M.w. substrates. The strong characteristic peaks of 4-aminothiophenol on the Ag-M.w.-*x* substrates were mainly centered at 1007, 1077, 1140, 1180, 1367, 1453, 1578 and 1650 cm^−1^ and the characteristic vibrational modes were given in Table 1 [37]. As shown in Figure 2a, the intensities of the Raman signal first increased with the increase of sputtering time and reached peak value at the time of 20 min, after which a marked decline of the Raman signal intensity was observed. The result indicated that the sputtering time of 20 min was the percolation threshold where the surface plasmon resonance was highly generated and dense “hot spots” could be significantly increased. Near the percolation threshold, the dense “hot spots” can be obtained, thus, amplifying the LSPR effect. It has been reported that the effective coupling effect of LSPR mostly occurred when the nanogaps between the noble metallic NPs were very small [38]. Therefore, we further stretched our experiments for the nanostructures of Au-Ag-M.w.-*x* to realize the coupling effect of two kinds of localized electromagnetic fields between Ag nanofilms and Au NPs. Hence, Au NPs with the average diameter of 30 ± 2 nm were deposited on the Ag-M.w.-*x* substrates to fabricate the Au-Ag-M.w.-*x* substrates. As shown in Figure 2b, the characteristic peaks of 10^−4^ M 4-aminothiophenol exhibited higher enhancement than those of Ag-M.w.-*x* hybrids. More importantly, the trend of the Raman signal enhancement was in agreement with that of Ag-M.w.-*x*. As shown in Table 2, from the increase factors we could conclude that the SERS enhanced effect of the Au-Ag-M.w.-*x* substrates was much higher than those of the Ag-M.w.-*x* substrates, especially for Au-Ag-M.w.-20 substrate. This active result can be attributed to the successful assembly of AuNPs on the Ag nanofilm. And the SERS substrate obtained the high enhancement with the assist of Au NPs which created high-density electromagnetic “hot spots”. Compared with the optimal SERS substrate (Cu-M.w.-30) in the previous report, the SERS substrates in our work is better than that one in terms of SERS enhancement. The SERS spectra of 10^−4^ M 4-aminothiophenol adsorbed onto Ag-M.w.-20 substrate, Au-Ag-M.w.-20 substrate, Cu-M.w.-30 substrate as well as 4-aminothiophenol solid powder were all exhibited in Figure 2c. Selected the characteristic band of 1578 cm^−1^ for example, as shown in Figure 2d, although the cost of Cu-M.w.-30 is lower than that of Ag-M.w.-20 and Au-Ag-M.w.-20 substrate, the SERS enhancement performance is visibly inferior to that of Ag and Au based SERS substrates. Especially for Au-Ag-M.w.-20 substrate, and the SERS increase factor is 26.40 (943,311 to 35,773) and 6.19 (943,311 to 133,369) higher than the Cu-M.w.-30 and Ag-M.w.-20 substrate.

### 3.2. Morphology Characterization

**Figure 3:**
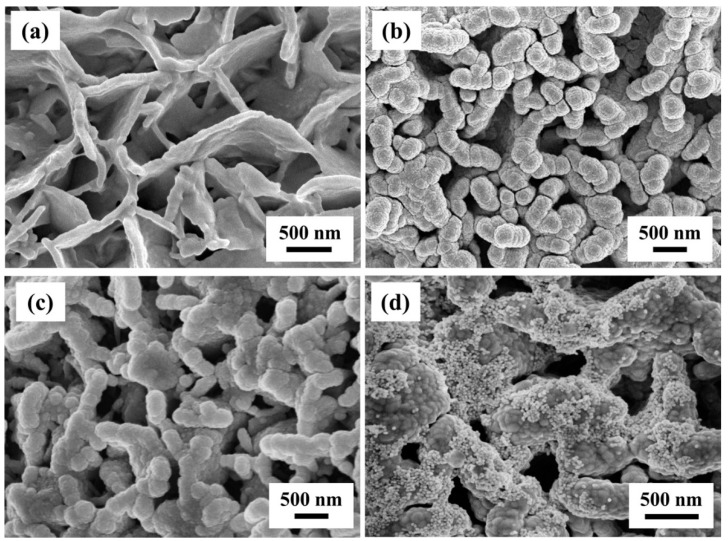
The FE-SEM images of M.w. before (**a**) and after 30 min Cu (**b**) and 20 min Ag (**c**) deposition; (**d**) The FE-SEM image of Au-Ag-M.w.-20 substrate.

**Figure 4:**
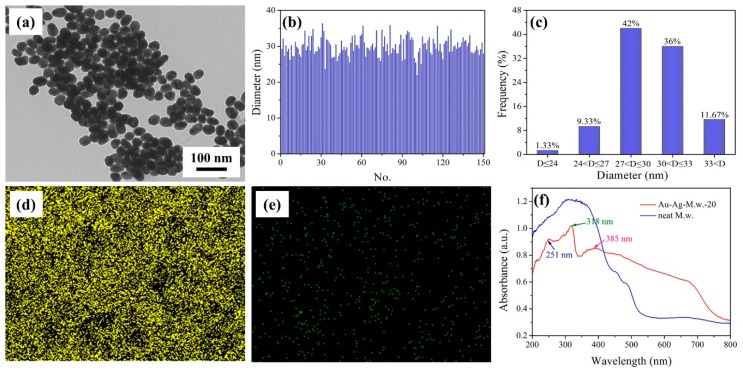
(**a**) TEM image of the Au NPs; The size (**b**) and size distribution (**c**) of the Au NPs; Elemental mapping of Ag (**d**) and Au (**e**) of Au-Ag-M.w.-20 substrate; (**f**) UV-vis spectra of Au-Ag-M.w.-20 and neat M.w. substrate.

The prepared flexible substrates were characterized by FE-SEM. As shown in Figure 3a, the surface of the M.w. was covered by a large number of randomly distributed irregular flake-like nanostructure. In addition, the image confirmed that these nanostructures consisted of vertical and interlaced nanoflakes whose size was ~100 ± 10 nm in width and 500–800 nm in length. These irregular distribution of nanostructures were linked together to form many nanoholes. This structural characteristics made the surface very rough and exhibited hydrophobic property. Generally, the novel irregular nanostructures can be regarded as natural plasmonic nanostructures if decorated with a thin layer of noble metal. Therefore, by using such hydrophobic M.w.s as bio-templates, we have designed and manufactured Cu-based SERS substrate in our previous report [34]. As shown in Figure 3b, by adjusting the sputtering time, the optimal Cu-M.w.-30 SERS substrate was obtained. The average width of the Cu nanofilm was about 300 ± 10 nm. To further optimize the M.w.-based plasmonic nanostructures, we continued to decorate the Ag nanofilm and Au NPs on this nanostructure. When the sputtering time was 20 min, the surface morphology was changed after Ag nanofilm coating. It could be obviously seen from Figure 3c that the vertical nanoflakes of Ag-M.w.-20 became thicker (~500 ± 10 nm) and the size of the nanohole therewith went down. The FE-SEM image of Au-Ag-M.w.-20 substrate was shown in Figure 3d, large-scale Au NPs were decorated on the surface of the Ag-M.w.-20 template, which ensured the Au-Ag-M.w.-20 substrate gain more appropriate nanogaps with high-density and multi-level “hot spots” for SERS performance. Figure 4a presents the TEM image of the Au NPs and the Au NPs were uniform in size. In order to better describe the Au NPs, the Nano Measurer software was applied to calculate the size and size distribution. As shown in Figure 4b, by counting 150 selected samples, the diameter of the Au NPs has a weak fluctuation. As the frequency distribution histograms presented in Figure 4c, the size distribution of Au NPs obeys the Gaussian distribution with the peak position centers at ~30 nm, which revealed that the average diameter of the Au NPs was 30 nm. In addition, the elemental mapping confirmed the successful decoration of Au NPs on the Ag-M.w.-20 surface. The elemental mappings of Ag (Figure 4d) and Au (Figure 4e) demonstrate a large-scale and uniform distribution of the Ag and Au elements. Enhanced UV-visible light absorption spectra of the Au-Ag-M.w.-20 and neat M.w. substrates were shown in Figure 4f. As we can see that the plasmon bands centered at ~251, 318 and 385 nm appeared. Although the wavelength of laser used in the Raman measurement (532 nm) is not matched with plasmon bands, the Au-Ag-M.w.-20 substrate exhibited strong Raman enhanced signals. This may be related to the rough indication in UV-vis absorption spectra of the matching between the excitation source and the surface plasmon resonance [27].

**Figure 5:**
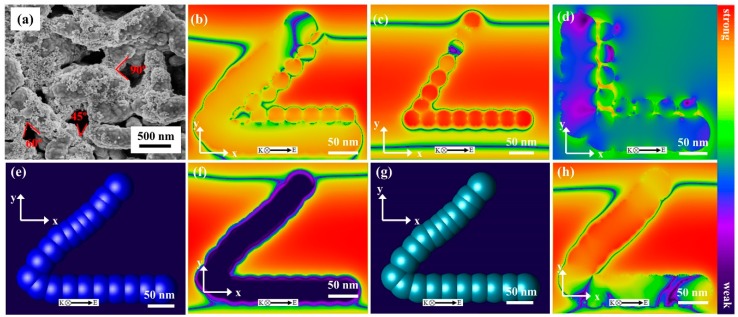
(**a**) FE-SEM image of Au-Ag-M.w.-20 substrate; (**b**–**d**) 3D-FDTD simulated electromagnetic field enhancement of crossed-nanofilms with the angle at (**b**) 45°, (**c**) 60° and (**d**) 90°, respectively; (**e**) and (**g**) 3D-FDTD models of Cu-M.w.-30 and Ag-M.w.-20 with an angle of 45°; (**f**) and (**h**) Spatial distributions of the electromagnetic field intensities around corresponding substrates.

Except for the rich collection effect resulted from the surface hydrophobicity, the types of noble metal materials and the LSPR effect excited near the nanostructure surface could also contribute to the electromagnetic field intensity [20]. Therefore, the 3D finite-difference time-domain (3D-FDTD) simulation method was introduced to simulate the intensity distribution of electromagnetic field. For the sake of accuracy, the length of the Ag nanofilm was set to be 300 nm, the diameter of the Au NPs was 30 nm and the nanogap between the Au NPs was 10 nm. Figure 5a exhibits the FE-SEM of the Au-Ag-M.w.-20 substrate, in which different angles (marked with red lines) could be observed. In this 3D-FDTD simulation, because the complex and irregular nanostructure is not easy to model and the Au NPs are very difficult to evenly distributed on the surface of Ag-M.w. substrates, we use this three typical angles (45°, 60° and 90°) to represent the nanostructure to study the electromagnetic field enhancement performance. Furthermore, a continuous square wave with a wavelength of 532 nm was selected as the incident light. We considered that the material of Au and Ag abided by Debye-Drude Model. Figure 5b–d show the calculated spatial distributions of electromagnetic field intensity for the Ag nanofilms crossed at 45°, 60° and 90°. The very strong electromagnetic field intensity was distributed almost at the nanogaps between the Au NPs and Ag nanofilms and a maximum EF of 62.18 V m^−1^ was obtained when the angle was 45°. In this case, the calculated EF was evaluated to be 1.49 × 10^7^. Meanwhile, the model and the distribution of electromagnetic field intensity of Cu-M.w.-30 and Ag-M.w.-20 nanofilms with the angles crossed at 45° was also shown in Figure 5e–h. Obviously, the SERS enhancement of Cu-M.w.-30 (EF = 2.24 × 10^4^) and Ag-M.w.-20 (EF = 8.07 × 10^5^) were weaker than Au-Ag-M.w.-20 SERS-active substrates. Simulation analysis shows that the types of noble metal material, size and nanofilm separation were crucial to efficiently tune the SERS performance. On the other hand, the novel flake-like nanostructure and the synergistic effects of Ag nanofilms and Au NPs would all contribute to the major enhancement of the electromagnetic field.

### 3.3. SERS Performances of the Prepared Substrate

**Figure 6:**
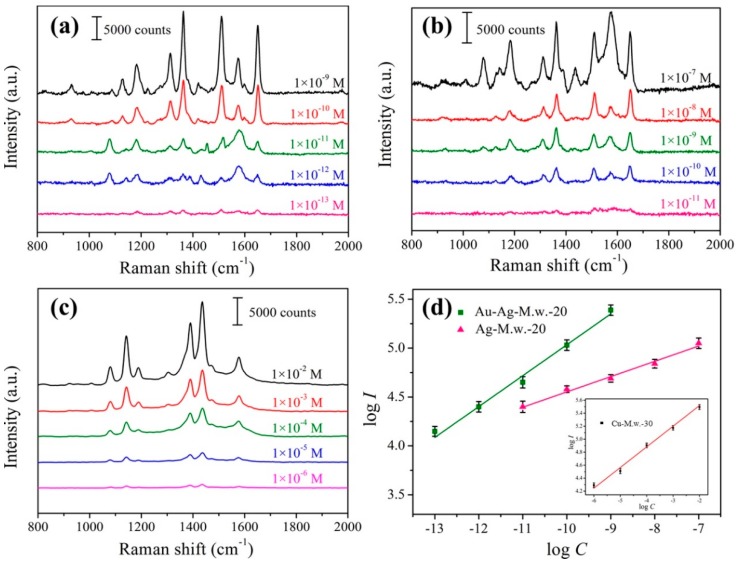
Raman spectra of 4-aminothiophenol with different concentrations recorded from Au-Ag-M.w.-20 (**a**), Ag-M.w.-20 (**b**) and Cu-M.w.-30 substrates (**c**); (**d**) linear calibration plot between the SERS intensities and 4-aminothiophenol concentrations in the logarithm scale of Cu-M.w.-30, Ag-M.w.-20 and Au-Ag-M.w.-20 substrates, respectively (the error bars were calculated based on 5 independent measurements).

To further investigate the SERS sensitivity of the Au-Ag-M.w.-20 substrate, we tested the Raman spectra of 4-aminothiophenol solutions with different concentrations (from 10^−9^ M to 10^−13^ M). For comparison, the sensitivity of Ag-M.w.-20 substrate was also determined. As shown in Figure 6a,b, the intensities of Raman peaks of 4-aminothiophenol decayed with the decrease of 4-aminothiophenol concentrations. The Raman characteristic peaks of 4-aminothiophenol could be evidently observed in the Figure 6a even at the concentration of 10^−13^ M. The LOD for Au-Ag-M.w.-20 substrate was two orders of magnitude lower than that of the Ag-M.w.-20 substrate. It indicated that the sensitivity of Au-Ag-M.w.-20 substrate was clearly superior to the Ag-M.w.-20 hybrid which allowed highly sensitive SERS detection. The better SERS behavior of Au-Ag-M.w.-20 substrate can be attributed to the successful decoration of Au NPs. After decorated the Ag nanofilms with Au NPs, the little probe molecules solution could certainly flow into the tiny nanogaps and could be more enriched around the “hot spots”, thus, generating more sensitive SERS signals. Meanwhile, in our previous report, the LOD for 4-aminothiophenol of Cu-M.w.-30 substrate was 10^−6^ M as shown in Figure 6c, which was 7 orders of magnitude higher than the Au-Ag-M.w.-20 substrate. This fully proves the superiority of the Au-Ag-M.w.-20 substrate in sensitive detection. The relationships between the SERS intensity at 1650 cm^−1^ and the 4-aminothiophenol concentration were plotted in the logarithm scale in Figure 6d, where the good linear relation could be found. The inset was the liner relationship at 1474 cm^−1^ of 4-aminothiophenol recorded by Cu-M.w.-30 substrates. The values of the correlation coefficient (R^2^) reached 0.988, 0.979 and 0.976 suggested that these types of substrates could all allow for the quantitative determination of the unknown concentrations of 4-aminothiophenol, but there is no doubt that the Au-Ag-M.w.-20 is the best candidate.

**Figure 7:**
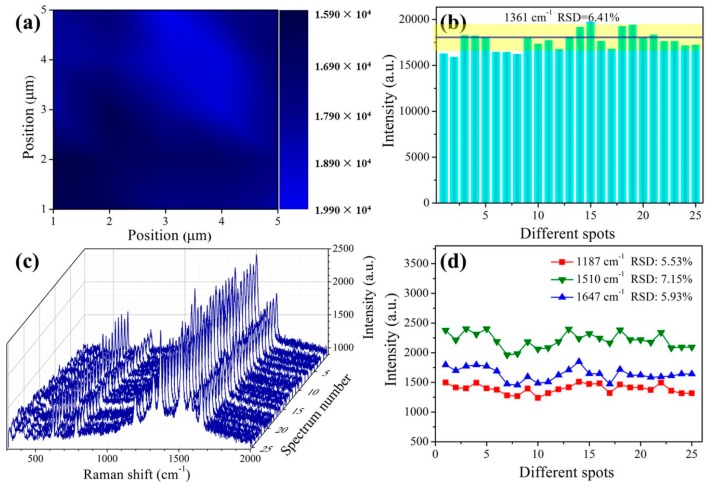
(**a**) the Raman mapping after the 10^−5^ M R6G dried on the Au-Ag-M.w.-20 substrate; (**b**) the main Raman vibrational intensities of 10^−5^ M R6G at the characteristic Raman peak of 1361 cm^−1^ (the height of the histogram represents the peak intensity); (**c**) SERS spectra of 10^−5^ M R6G obtained from 25 randomly selected spots on 5 Au-Ag-M.w.-20 substrate; (**d**) the peak intensities of Raman band at 1187, 1510 and 1647 cm^−1^ and the corresponding RSD values (the value of the ordinate is the peak intensity).

**Figure 8:**
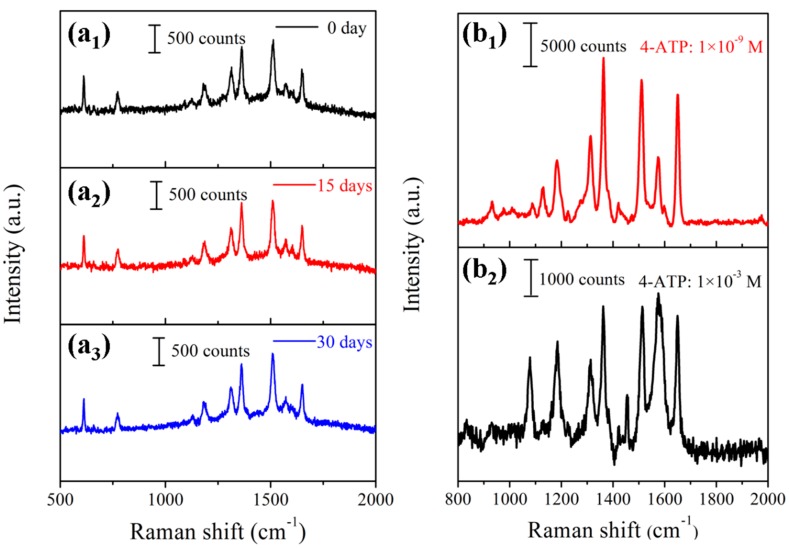
SERS signals of R6G with the concentration of 10^−5^ M on the Au-Ag-M.w.-20 substrates under the exposure to the ambient temperatures for 0 day (**a_1_**), 15 days (**a_2_**) and 30 days (**a_3_**); (**b_1_**) SERS spectrum of 10^−9^ M 4-aminothiophenol solution acquired from Au-Ag-M.w.-20 substrate; (**b_2_**) normal Raman spectrum of 10^−3^ M 4-aminothiophenol solution.

In addition to the high sensitivity, the reproducibility of the Raman signals is a crucial parameter which affects the reliability in application. In general, the RSD value of the Raman signal intensity is usually calculated to evaluate the reproducibility of a SERS substrate. It has been widely confirmed that a RSD value of the SERS measurement less than 20% over micrometer-sized area indicated high reproducibility [39]. Therefore, the SERS mapping analysis was experimented on the Au-Ag-M.w.-20 substrates and the obtained spectra were with 1 μm/point scan step and covered an area of 5 × 5 μm^2^. As shown in Figure 7a, the color bar on the right was proportional to the peak areas at 1361 cm^−1^ for 10^−5^ M R6G. Figure 7b shows the Raman signal intensity distribution of 25 points of the 1361 cm^−1^. The RSD value was calculated to be 6.41% according to the following Equation (1) [40]:

(1) $$RSD=\frac{\sqrt{\frac{\sum_{i=1}^{n}{\left(I_{i}-\bar{I}\right)}^{2}}{n-1}}}{\bar{I}}$$

where the $\bar{I}$ represents the average intensity of the Raman signal, *n* is 25 in agreement with the number of the measured spectrum, $I_{i}$ is the Raman intensity of each spectrum at the 1361 cm^−1^. Meanwhile, the substrate-to-substrate reproducibility was also measured at 25 randomly chosen points from 5 Au-Ag-M.w.-20 substrates. As shown in Figure 7c, the 25 spectra obtained from different points overlapped very well. The RSD values of three characteristic peaks at 1187, 1510 and 1647 cm^−1^ were calculated to be 5.53%, 7.15% and 5.93%, as shown in Figure 7d. The results fully indicated that the SERS substrate has outstanding reproducibility across the entire surface, which is likely to benefit from the dense distribution of the Au NPs and the homogenous Ag nanofilms coating on the SERS substrates. Furthermore, stability is another parameter for evaluating the SERS-active substrate. Therefore, the stability of the Au-Ag-M.w.-20 substrates was tested by the detection of R6G with the concentration of 10^−5^ M. The Raman spectra of R6G from Au-Ag-M.w.-20 substrate for different detection times were displayed in Figure 8a. By calculating the peak intensity of 1610 cm^−1^, the Raman signal intensity fell only by 10.59% within 30 days. In consequence, our Au-Ag-M.w.-20 substrate possesses outstanding reproducibility well antioxidant stability compared to Cu-M.w.-30 substrate [34] which can be used in the practical application.

### 3.4. EF Calculation

Enhancement factor (EF) was calculated to further quantify the enhancement distribution of the Au-Ag-M.w.-20 substrate according to the following equation [41]:

(2) $$EF=\left[\frac{I_{SERS}}{I_{Raman}}\right]\times \left[\frac{N_{Raman}}{N_{SERS}}\right]$$

where the *I_SERS_* and *I_Raman_* represent the integrated peak intensity of the SERS signal and the normal Raman signal, *N_SERS_* and *N_Raman_* are the number of the probe molecules on the substrates within the laser spot. Here, we chose the 10^−3^ M 4-aminothiophenol solution on the silicon wafer as the Raman substrate, while SERS substrate was used the Au-Ag-M.w.-20 hybrid with 10^−9^ M 4-aminothiophenol. Besides, the Raman characteristic band of 1367 cm^−1^ was chosen to calculate the integrated *I* values. As shown in Figure 8b_1_,b_2_, the value of *I_SERS_* and *I_Raman_* calculated to be 3.40 × 10^5^ and 8.56 × 10^4^, respectively. Therefore, the ratio *I_SERS_*/*I_Raman_* was equaled to ~3.98. The *N_Raman_* and *N_SERS_* can be calculated as followed [42]:

(3) $$N=\left(\frac{N_{A}\times M\times V_{solution}}{S_{sub}}\right)\times S_{laser}$$

where *N_A_* and *M* are the Avogadro constant and molar concentration of the solution, *V_solution_* stands for the volume of the droplet of 4-aminothiophenol, *S_sub_* denotes the size of the 4-aminothiophenol solution on the different substrates and *S_laser_* is the size of the laser spot, respectively. In the Raman setup, the laser diameter was 1 μm, so the *S_laser_* equaled 0.785 μm^2^ and the 4-aminothiophenol drop area on the silicon wafer is approximately 1.2-time larger than that on SERS substrate. Thus, the ratio of *N_Raman_*/*N_SERS_* was equaled to 8.3 × 10^5^. Therefore, the SERS EF value of Au-Ag-M.w.-20 substrate at the 1367 cm^−1^ band was ~3.30 × 10^6^.

### 3.5. Collection of Pesticide Residues by Au-Ag-M.w.-20 Substrate

**Figure 9:**
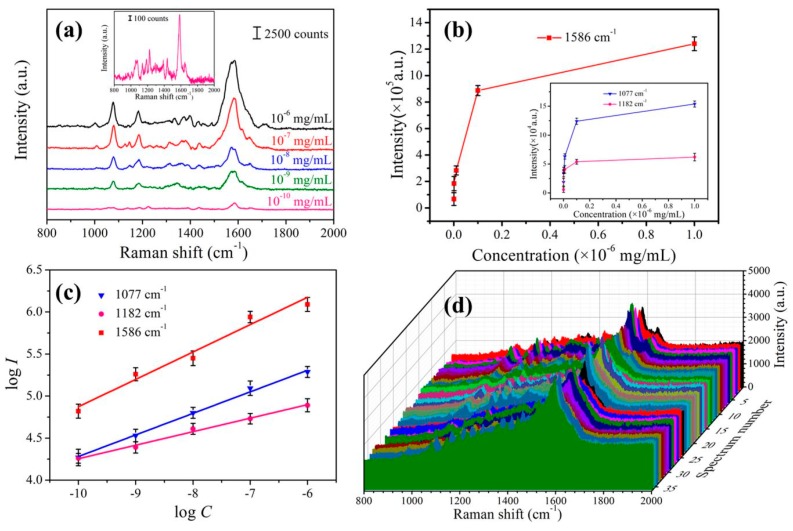
(**a**) SERS spectra of cypermethrin with different concentrations collected from apple peels; (**b**) Corresponding SERS intensities of main peaks at different concentrations; (**c**) Linear calibration plot between SERS intensity and cypermethrin concentration; (**d**) SERS spectra of 10^−8^ mg/mL cypermethrin collected from 6 apple peels samples by using the Au-Ag-M.w.-20 substrate.

Multifarious pesticide residues are attracting more and more attention due to the potential harm to human health. Serious overdose of pesticide residues can cause acute intoxication, while mild pesticide residues chronically accumulated in human body can also cause many chronic diseases. At present, the widely used methods for pesticide residue detection are liquid chromatography [43], gas chromatography mass spectroscopy [44] and fluorescence analysis [45]. The accuracy of these methods is very high, but the pretreatment processes are very tedious, which is difficult to meet the rapid and on-spot detection in fruit and vegetables. Therefore, it is an urgent problem to investigate a rapid sampling and facile pesticide residue detection technology. To evaluate the real-world application of the Au-Ag-M.w.-20 substrate, cypermethrin was detected to test the SERS practicability of this bio-inspired substrate. The cypermethrin has been widely used in the planting process of the tea, fruits and vegetables. Once the exposure is large, it can cause headache, nausea, vomiting, coma and even shock. Therefore, developing a facile and rapid approach to sensitively and on-spot detect cypermethrin is necessary. The detailed detection of cypermethrin from apple peels was performed as described in the experimental section. Figure 9a shows the Raman spectra of cypermethrin ranging from 10^−6^ mg/mL to 10^−10^ mg/mL. The Raman peaks observed at 1077, 1182 and 1586 cm^−1^ were the fingerprint peaks of cypermethrin [46]. The Raman signals could still be observed from the inset of Figure 9a even when the concentration fell to 10^−10^ mg/mL, indicating highly sensitive detection capability of Au-Ag-M.w.-20 substrate. Figure 9b,c shows the relationship between the integrated Raman signal intensities and the concentrations of cypermethrin. Obviously, as the concentration decreased, the peak intensity gradually decreased. In this case, there were linear relationships at 1077, 1182 and 1586 cm^−1^, which indicated the excellent quantitative evaluation of the cypermethrin as shown in Figure 9c. To further evaluate the reproducibility of Au-Ag-M.w.-20 substrate in detecting of cypermethrin, the SERS spectra of 10^−8^ mg/mL cypermethrin were shown in Figure 9d, revealing high reproducibility of the Raman signal.

**Figure 10:**
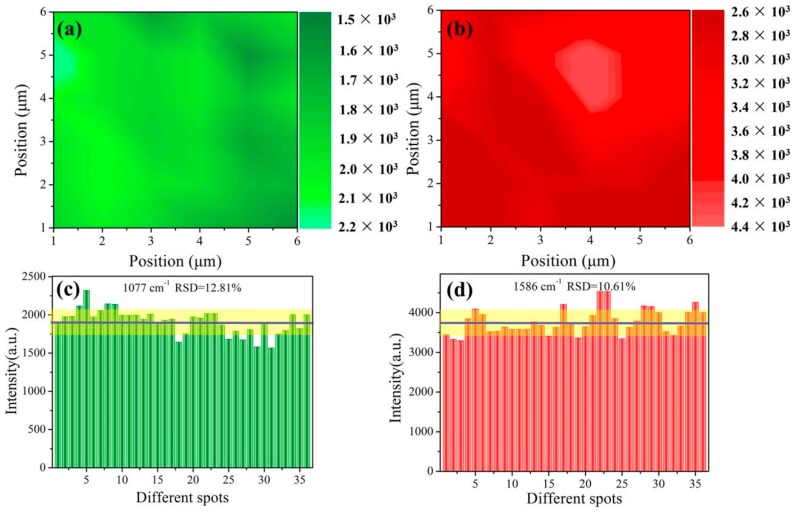
SERS mapping of the peaks across a 6 × 6 µm^2^ area measured at: (**a**) 1077 cm^−1^ and (**b**) 1586 cm^−1^ for cypermethrin; (**c**,**d**) the main Raman vibrational intensities of 10^−8^ mg/mL cypermethrin at characteristic Raman peaks (c: 1077 cm^−1^; d: 1586 cm^−1^).

In addition, a 6 × 6 µm^2^ area on a random Au-Ag-M.w.-20 substrate was selected to test the SERS behaviors and the results were displayed in Figure 10a,b. The relatively uniform color distribution suggested that the outstanding step-to-step reproducibility of our Au-Ag-M.w.-20 substrates. Figure 10c,d quantitatively exhibited the intensity variation of 36 spots at 1077 and 1586 cm^−1^, respectively. Correspondingly, the RSD values were calculated to be 12.81% and 10.61% according to Equation (1). These results adequately revealed that the bio-inspired Au-Ag-M.w.-20 substrate possesses high practicality as well as credibility in real detection.

## 4. Conclusions

In summary, we have successfully fabricated a kind of ultra-sensitive and flexible SERS platform based on the irregular mantis wing nanoarray by magnetron sputtering technique and physical deposition method. With the 4-aminothiophenol as probe molecules, the Au-Ag-M.w.-20 SERS-active substrate exhibited the most remarkable SERS performance compared with single Cu or Ag nanofilms based hybrids, which could be ascribed to the large-scale “hot spots” that existed between the appropriate nanogaps. In addition, the Au-Ag-M.w.-20 substrate could achieve sensitive SERS measurements with a linear range of the detection of 4-aminothiophenol and the signal reproducibility for R6G was well within 5.53%–7.15% variations. A practical application for the detection of cypermethrin on apple peels was conducted with a LOD reached to be 10^−10^ mg/mL. Therefore, the naturally inspired substrate showed high potential in real-life detection, which was expected to find application in food safety and environmental monitoring.

## Figures and Tables

**Table 1:** Experimental assignments of vibrations of 4-aminothiophenol on the SERS substrate (*ν*, stretching; *δ*, bending; *γ*, out-of-plane deformation (respect to the benzene ring)).

Modes	SERS 532 nm (cm^−1^)	Band Assignment
18a(*a_1_*)	1007	*γ_CC_ + γ_CCC_*
7a(*a_1_*)	1077	*ν_CS_*
9b(*b_2_*)	1140	*δ_CH_*
9a(*a_1_*)	1185	*δ_CH_*
14b(*b_2_*)	1313	*ν_CC_ + δ_CH_*
3b(*b_2_*)	1367	*ν_CC_ + δ_CH_*
19b(*b_2_*)	1453	*ν_CC_ + δ_CH_*
8b(*b_2_*)	1578	*ν_CC_*
-	1650	*δ_CH_*

**Table 2:** Raman intensities of the peak at 1185 cm^−1^.

Sputtering Time (min)	Peak Intensity *I* (count)		Increase Factor (*I*_Au-Ag-M.w.-*x*_/*I*_Ag-M.w.-*x*_)
	*I*_Au-Ag-M.w.-*x*_	*I*_Ag-M.w.-*x*_	
10	106,148 ± 1542.6	84,685 ± 748.8	1.25 ± 0.025
20	263,045 ± 1146.4.3	125,620 ± 1640.5	2.09 ± 0.032
30	175,894 ± 1320.3	105,630 ± 990.0	1.67 ± 0.017
40	157,304 ± 1114.3	42,583 ± 909.7	3.69 ± 0.097
